# Genetic Diversity in *FUB* Genes of *Fusarium oxysporum* f. sp. *cubense* Suggests Horizontal Gene Transfer

**DOI:** 10.3389/fpls.2019.01069

**Published:** 2019-09-04

**Authors:** Siwen Liu, Bo Wu, Shuxia Lv, Zongzhuan Shen, Rong Li, Ganjun Yi, Chunyu Li, Xiuwu Guo

**Affiliations:** ^1^College of Horticulture, Shenyang Agricultural University, Shenyang, China; ^2^Key Laboratory of South Subtropical Fruit Biology and Genetic Resource Utilization, Ministry of Agriculture, Key Laboratory of Tropical and Subtropical Fruit Tree Research of Guangdong Province, Institution of Fruit Tree Research, Guangdong Academy of Agricultural Sciences, Guangzhou, China; ^3^College of Bioscience and Biotechnology, Shenyang Agricultural University, Shenyang, China; ^4^Jiangsu Key Lab for Solid Organic Waste Utilization, Key Laboratory of Plant Nutrition and Fertilization in Low-Middle Reaches of the Yangtze River, Ministry of Agriculture, College of Resources and Environmental Sciences, Nanjing Agricultural University, Nanjing, China

**Keywords:** Panama disease, *Fusarium oxysporum* f. sp. *cubense*, fusaric acid, horizontal gene transfer, phylogeny

## Abstract

Fusaric acid (FA) is an important secondary metabolite of many Fusarium species and involved in the wilt symptoms caused in banana by *Fusarium oxysporum* f. sp. *cubense (Foc)*. To investigate the evolution characteristics of the 12 *Foc* FA biosynthetic genes (*FUB*), coding sequences of the 12 *FUB* genes and three housekeeping genes, *EF-1*α*/RPB1/RPB2* (translation elongation factor-1α/RNA polymerase II subunit I/RNA polymerase II subunit II), were subjected to genetic diversity analysis, phylogenetic analysis, recombination detection, and selective pressure analysis. The results of selective pressure analysis showed that the 15 genes were mainly subjected to negative selection. However, a significantly higher number of silent mutations, which could not be simply explained by selective pressure difference, were observed in the 12 *FUB* genes in *Foc* than in the three housekeeping genes. Infraspecies phylogeny and recombination detection analysis showed that significantly more horizontal gene transfer (HGT) events (normalized) had occurred in the *FUB* genes than in the three housekeeping genes. In addition, many of these events involved outgroup isolates and significantly increased the genetic diversity of *FUB* genes in *Foc*. The infraspecies phylogenetic analysis suggested that the polyphyletic phylogeny proposed for Foc requires further discussion, and the divergence of race 1, race 4, and the common ancestor of several *F. oxysporum* (*Fo*) isolates pathogenic to nonbanana plants should have diverged over a short period. Finally, our results suggest that the *FUB* genes in *Fo* should have benefited from HGT to gain a relatively high genetic diversity to respond to different host plants and environments despite mainly being subject to negative selection.

## Introduction

*Fusarium oxysporum* (*Fo*) f. sp. *cubense* (*Foc*), the causal agent of Fusarium wilt of banana (*Musa* spp.), which is also known as Panama disease, is the most important soil-borne pathogen limiting banana production in the world (Ploetz, 2015). Like other *Fusarium* f. sp., *Foc* produces many toxic secondary metabolites; among these, fusaric acid (FA) is one of the most studied mycotoxins (Desjardins and Proctor, 2007; Bohni et al., 2016; Singh et al., 2017; López-Díaz et al., 2018) and a host-nonspecific toxin. FA is involved in the toxicity of *Fusarium* spp. towards plants, animals, and human beings (Ghazi et al., 2017; Singh et al., 2017; López-Díaz et al., 2018) and in interactions with environmental microorganisms (Brown et al., 2015; Bohni et al., 2016; Simonetti et al., 2018). FA has been reported to be phytotoxic to different plants involving multiple biological processes. For instance, it has been reported to chelate metal ions inside tomato (López-Díaz et al., 2018), disturb the water balance in cucumber (Wang et al., 2015), and cause programmed cell death in tobacco suspension cells (Jiao et al., 2013). In Panama disease, FA production by *Foc* is involved in disturbing the water balance and causing the wilt symptom (Dong et al., 2012; Li et al., 2013; Dong et al., 2014) and has been shown to be essential for its virulence on banana plantlets (Ding et al., 2018).

Due to the important function of FA in many *Fusarium* spp., much attention has been paid to the genes involved in its biosynthetic and related regulatory processes (Brown et al., 2012; Niehaus et al., 2014; Brown et al., 2015; Studt et al., 2016; Ding et al., 2018). Like many other secondary metabolite biosynthetic genes, which are generally located in gene clusters (Hoogendoorn et al., 2018), a *FUB* gene cluster consisting of 12 genes (*FUB1* to *FUB12*) was identified in different *Fusarium* species (Brown et al., 2015; Studt et al., 2016). The functions of the *FUB* genes in *Fusarium* were predicted through the functions of their annotated homologs, including a polyketide synthase (PKS, *FUB1*), an unknown protein (*FUB2*), an aspartate kinase (*FUB3*), a serine hydrolase (*FUB4*), a homoserine *O*-acetyltransferase (*FUB5*), an NAD(P)-dependent dehydrogenase (*FUB6*), an *O*-acetylhomoserine (thiol-)lyase (*FUB7*), a nonribosomal peptide synthetase (NPRS)-like enzyme (*FUB8*), an FMN-dependent dehydrogenase (*FUB9*), two C6 transcription factors (*FUB10* and *FUB12*), and a major facilitator superfamily transporter (*FUB11*) (Studt et al., 2016). FA production is differentially altered in deletion mutants of each *FUB* gene (Brown et al., 2015; Studt et al., 2016), suggesting that the importance of the 12 genes is different. *FUB1* is the key gene in the *FUB* gene cluster, and FA production was abolished or reduced by 95% in the *Fo FUB1* deletion mutant (Brown et al., 2015; López-Díaz et al., 2018). FA production was abolished in deletion mutants of *FUB10* in *Fusarium verticillioides* (*Fv*), *Fusarium fujikuroi* (*Ff*), and *Fo*, and *FUB10* has been proven to be a transcription factor positively regulating the transcription of other *FUB* genes (Brown et al., 2015; Studt et al., 2016). Deletion mutants of the other transcription factor-encoding gene, *FUB12*, exhibited incomplete loss of FA production in *Fv* and *Ff* (Brown et al., 2015; Studt et al., 2016). *Fo* deletion mutants of *FUB3*, *FUB6*, or *FUB8* also abolished FA production, but in corresponding *Fv* mutants, FA production was reduced to only a certain extent (Brown et al., 2015). A *FUB4* deletion mutant of a *Foc* race 4 strain lost its capacity for FA production and was significantly weakened in its virulence towards banana (Ding et al., 2018).

The infraspecies phylogeny of *Foc* has been suggested to be complex. Three races of *Foc* that are pathogenic to different host banana types have been reported: race 1, affecting Gros Michel (AAA) and some AAB or ABB bananas; race 2, pathogenic to ABB cooking bananas; race 4, affecting Cavendish bananas; and race 1 and race 2 suspects (Mostert et al., 2017). Race 4 isolates were further divided into two groups according to their different requirements of disease-predisposing conditions: tropical race 4 (TR4) and subtropical race 4 (STR4) (Ploetz, 2015; Ploetz et al., 2015). According to vegetative compatibility, *Foc* isolates were assigned to 24 vegetative compatibility groups (VCGs) (Fourie et al., 2009). *Foc* was suggested to have multiple evolutionary origins based on different nuclear and mitochondrial genes (O’Donnell et al., 1998; Fourie et al., 2009; Czislowski et al., 2018). In the study of Fourie et al. (2009), polyphyletic phylogeny was supported for not only *Foc* but also race 1 and race 4, and the same sets of VCGs were generally clustered together. An infraspecies phylogeny analysis based on three housekeeping genes (*EF-1*α/*RPB1*/*RPB2*) supported monophyletic phylogeny of race 1 VCGs and race 4 VCGs (Czislowski et al., 2018). However, significant discordance was observed between some of the phylogenetic trees based on the *SIX* (Secreted In Xylem) genes and the infraspecies tree, suggesting horizontal gene transfer (HGT) events in the *SIX* genes in *Foc* (Czislowski et al., 2018). The study of Maryani et al. (2019) based on *EF-1*α/*RPB1*/*RPB2* also suggested that the *Foc* TR4 group should be monophyletic, but *Foc* race 1 isolates were clustered into multiple lineages. Horizontal gene transfer rather than convergent evolution was suggested to explain the polyphyletic phylogeny of race 1 and race 4 observed (Czislowski et al., 2018), and it was also suggested to have contributed to the high diversity level observed in race 1 compared with TR4 (Maryani et al., 2019). According to the infraspecies analysis in the study of Czislowski et al. (2018), some *Fo* f. sp. isolates and nonpathogenic *Fo* isolates should have originated from *Foc* race 1. *Foc* has no known sexual cycle and is supposed to undergo sexual production only rarely, if at all (Kerényi et al., 2004; Ploetz, 2015), and the mechanism of HGT in *Foc* remains unknown.

As stated above, FA production plays an important role in pathogen plant/environmental microorganism interactions in many *Fusarium* (sub)species including *Foc*. Considering its specific function, we hypothesize that the conservation of protein function (negative selection) should be the main selective force on *Foc FUB* genes when the host and environment are relatively stable, but selection of new advantageous mutations (positive selection) should also be possible if the fungus is adapting to a new host or environment. Based on coding sequences (CDSs) of the 12 *FUB* genes and three housekeeping genes from 33 *Foc* isolates, this study aimed to reveal the genetic diversity and evolutionary characteristics including nonsynonymous/synonymous substitution ratio and HGT in *FUB* genes which are largely determined by the selective forces. And in the meantime, an effort was also made to infer the infraspecies phylogeny of *Foc*.

## Materials and Methods

### Isolates and Group Assignments

Whole-genome sequences of 33 *Foc* isolates (**Table 1**) collected from multiple countries in South Asia, South Africa, and America were obtained from the *Foc* genome sequencing project (data unpublished). Published genomes of five *Fo* isolates, II5 (NRRL#54006, GCA_000260195.2, belonging to *Foc* race 4), Fo25433 (NRRL#54006, GCA_000260175.2), Fo47 (NRRL#54002, GCA_000271705.2), Fo4287 (NRRL#34936, GCA_000149955.2), and Fo26406 (NRRL#26406, GCA_002318975.1), and an outgroup *Fv* isolate, Fv7600 (GCA_000149555.1), were downloaded from the National Center for Biotechnology Information (NCBI) assembly database. The Northern Regional Research Laboratory (NRRL) codes were assigned by the Agricultural Research Service Culture Collection, US Department of Agriculture, and the IDs following the NRRL codes in the brackets are GenBank accession numbers. A total of 16 race 1 isolates from the *Foc* genome sequencing project were assigned to R1, including two nonpathogenic *Fo* isolates JB255 and JB553, which should belong to R1 according to phylogenetic analysis; and 18 race 4 isolates, including 17 from the *Foc* genome sequencing project and II5, were assigned to R4. Fo4287, Fo47, and Fo26406, which formed a monophyletic group in the phylogenetic analysis in this study (**Figures 2A, B**), were assigned to the 3Fo group. Fo25433 was not assigned to the R1 group considering its special identity, although it should have originated from R1 according to phylogeny analysis.

**Table 1:** Information of sequenced *Fo* isolates.

Group	Strain	VCG*	Host banana cultivar	Sampling location
R1	Race1-CAV2013	VCG0128	Chuoi tay cao	Van Giang, Vietnam
R1	Race1-22994	VCG0128	Bluggoe	South Johnstone, Australia
R1	Race1-188	VCG01212	Ney Poovan	Tenguero Station, Tanzania
R1	Race1-623	VCG01220	Williams	Carnarvon, Australia
R1	Race1-871	VCG01217	Pisang Rastali	Malaysia
R1	Race1-939	VCG0123	Kluai Namwa	Thailand
R1	Race1-967	VCG0124/5	Latundan	Philippines
R1	Race1-MW2	VCG01214	Harare	Misuku, Malawi
R1	Race1-Mal6	VCG01217	Pisang Rastali	Kg. Taboh, Malaysia
R1	Race1-1983	VCG0123	Pisang Awak	Taiwan
R1	Race1-24223	VCG1220	Williams	Carnarvon, West Australia
R1	Race1-GD01	VCG01220	Pisang Awak	Guangdong, China
R1	Race1-HN05	VCG0123	Gros Michel	Hainan
R1	Race1-MW40	VCG01214	Harare	Misuku, Malawi
R1	JB255	–^#^	Non-pathogenic, soil	Kiepersol, South Africa
R1	JB553	–	Non-pathogenic, soil	Kiepersol, South Africa
R4	TR4-CAV2318	VCG0121	Namwa	Kuosin E, Taiwan
R4	TR4-CAV300	VCG01213	Valery	Southeast Sumatra, Indonesia
R4	TR4-STSUM5	VCG01213	Pisang Batan	Sumatra, Indonesia
R4	TR4-HN17	VCG01216	Cavendish	Hainan, China
R4	TR4-Mal2a	VCG01216	Pisang Raja	MARDI, Selangor, Malaysia
R4	STR4-105	VCG0120	Cavendish	Kiepersol, South Africa
R4	STR4-612	VCG01215	Gros Michel	Costa Rica
R4	STR4-SH3142	VCG01211	SH3142	Queensland, Australia
R4	STR4-980	VCG0120/15	–	Canary Island
R4	STR4-Pacovan	VCG0120	Pacvan	Bahia, Brazil
R4	STR4-187	VCG01210	Apple	Florida, USA
R4	STR4-195	VCG01219	Pisang Ambon	Indonesia
R4	STR4-618	VCG0122	Cavendish	Philippines
R4	STR4-M3	VCG01210	Gros Michel	FHIA Villa Clara, Cuba
R4	STR4-II12	VCG01219	Pisang raja garing	Cibinong Collection, Indonesia
R4	STR4-GD26	VCG0126	Pisang Awak	Guangdong, China
R4	STR4-1089	VCG0129	Lady finger	Cooloolabin, QLD, Australia

*VCG, abbreviation of vegetative compatibility group. #The short dash line indicates the information is unknown or un-recorded.

**Figure 1:**
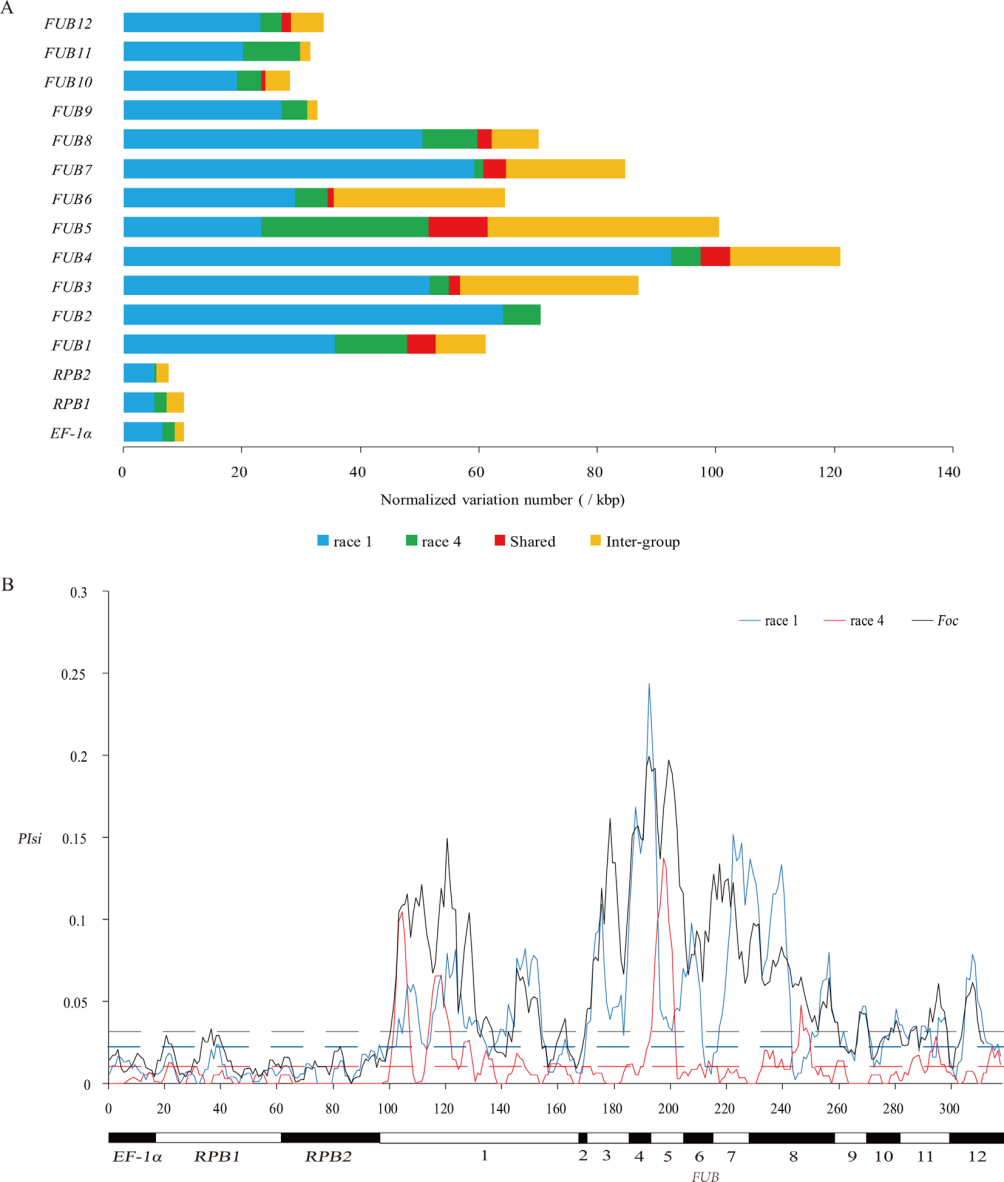
Genetic diversity of 34 *Foc* isolates based on 15 genes. **(A)** Distribution of intragroup and intergroup variations in *Foc* isolates based on 15 genes. Blue and green bars indicate the number of intragroup variations specific in R1 and R4, respectively. The red bar indicates the amount of intragroup variation shared by R1 and R4 isolates. Yellow bar indicates the number of intergroup variations bet ween R1 and R4. **(B)** Distribution of *PIsi* on windows of 100 silent sites across the 15 genes. The horizontal dashed lines indicate the threshold *PIsi* value, which was significantly (*p* < 0.01) higher than the *PIsi* level of the three housekeeping genes in R1, R4, and *Foc*.

**Figure 2:**
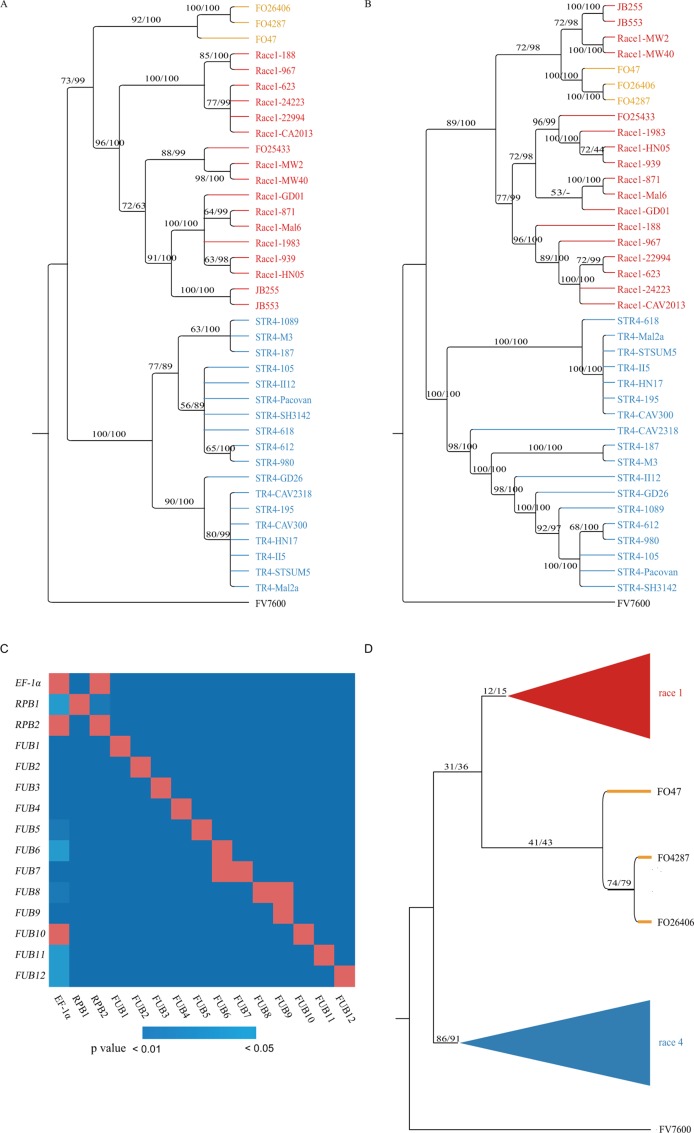
*Foc* infraspecies phylogeny inference and discordance between phylogenetic trees based on different genes. **(A** and **B)** Phylogenetic trees constructed based on concatenated sequences of *EF-1*α*/RPB1/RPB2* and the 12 *FUB* genes, respectively. The support values on the branches are ML bootstrap proportions/BI posterior probabilities. R1, R4 and 3FO isolates were denoted by red, blue, and orange colors respectively. **(C)** SH test of the tree concordance between different genes. The best ML tree based on genes listed on the vertical axis was tested using the best tree and sequence of the gene listed on the horizontal axis. The color of the squares was determined by the p value of the SH test: blue indicated significance, and red indicated nonsignificance (p > 0.05). **(D)** Infraspecies tree inferred using the BSRRSS method. The numbers on branches are the support rates of the branch in 1,000 randomly sampled 1-kbp/2-kbp gene segments.

### Sequence Alignment and Genetic Diversity Analysis

CDSs of *EF-1α*, *RPB1*, *RPB2*, and 12 *FUB* (*FUB1–12*) genes were obtained from the reference genome sequence of *Foc* race 4 isolate II5 (GenBank accession no. from MH972571 to 973155) (Guo et al., 2014). Then, the CDSs of the 15 genes were pulled out from all the genomes according to the results of local BLASTN alignment using II5 CDSs as queries, and no highly similar (>95% nucleotide similarity) paralog was identified for any gene. Multiple sequence alignment for each gene and concatenated sequences of *EF-1α/RPB1/RPB2*, the *FUB* gene cluster and the 15 genes was carried out using Clustal X version 2 (Larkin et al., 2007). Some of the CDSs were slightly different (probably due to different gene models applied in genome annotations or mutations causing transcript alteration that could not be distinguished and should not be a problem in this study) and were adjusted using their genome sequences to accommodate the CDS of the *Foc* II5 isolate.

Parameters, including *Pi*, the number of intragroup/intergroup variations, the number of synonymous/nonsynonymous mutations, the number of haplotypes, Dxy (the average number of nucleotide substitutions per site between populations), and the haplotype diversity of R1, R4 and *Foc* were calculated for each gene using DnaSP v6.12.01 (Rozas et al., 2017). Some of the mutations occurred on the same codons and could be assigned to be either non-synonymous or synonymous, depending on their occurrence orders. A total 9 such mutations were not assigned to either class. Silent loci in CDSs are generally subjected to neutral evolution in the genome, and the synonymous substitution rates among different genes have been shown to match a uniform point mutation rate across genes in *Escherichia coli* (Maddamsetti et al., 2015). A high level of synonymous mutations could be utilized as a potential signal of HGT (Proctor et al., 2013; Chen et al., 2018). Because the three housekeeping genes were mainly subjected to negative selection, which would not induce loss of *PIsi*, the *PIsi* level in these three genes was utilized as the control level to detect regions with significantly increased *PIsi*. *PIsi* was calculated in 100 silent site windows with a step size of 25 across the concatenated sequences of the 15 genes in R1, R4 and *Foc* using DnaSP v6.12.01. The first 97 windows were located within the three housekeeping genes and used as controls. The 99% cumulative probability threshold *PIsi* value was calculated for R1, R4 and *Foc* based on the normal distribution parameters calculated from the 97 windows in EXCEL, and *PIsi* values higher than this threshold are supposed to be significantly (p < 0.01) higher than those for the three housekeeping genes.

### Infraspecies Phylogenetic Analysis

With the aligned sequences, jModelTest-2.1.10 (Darriba et al., 2012) was applied to choose the best models for phylogenetic tree construction based on ML and BI. Then, PhyML 3.1 (Guindon et al., 2010) was applied to produce ML trees using the best-fit model for concatenated sequences of *EF-1α/RPB1/RPB2* and the *FUB* gene cluster and individual genes with 1,000 bootstrap replicates, and MrBayes v3.2 was used for tree construction by BI (Ronquist et al., 2012). Partitioned analysis unlinking models and parameters among different gene loci was applied on concatenated sequences by MrBayes v3.2. The SH test (Shimodaira and Hasegawa, 2001) was implemented in RAxML v8.2.12 (Stamatakis, 2014) to determine whether there was a significant difference in the tree topologies supported by different genes.

When concatenated gene sequences were used in phylogenetic inference, large regions of weak phylogenetic signals (produced when three or more species diverged in a short time) that support the true species tree topology could be obscured by short regions of strong phylogenetic signals caused by recent HGT events, and in this case, the standard nonparametric bootstrap could have lost its power (Heled and Drummond, 2010). In theory, the introgressed genetic material should account for a smaller proportion of the genome than the original species/infraspecies. Accordingly, the BSRRSS method was carried out by our Python script by calculating the branch support rate of branches in the best trees (obtained from 500 independent ML tree searches using RAxML v8.2.12) constructed for 1,000 randomly sampled 1 or 2 kbp gene segments from the concatenated sequence. A majority rule extended consensus tree was obtained from the 1000 best trees using RAxML v8.2.12, which should be a more reliable species tree. The low support rate of inner-group branches could have derived from either HGT or low sequence difference; thus, our main attention would be focused on the splitting of *Fo* groups. In theory, it would be more reasonable to sample an equal number of gene segments from each ‘unlinked’ locus (at most four in this study, *EF-1α, RPB1, RPB2* and the *FUB* gene cluster), but considering that the gene sequences of the three housekeeping genes were short, it was not applied in this study.

### Recombination Detection

Recombination detection was carried out using 7 different algorithms implemented in RDP v.4.95 (Martin et al., 2015), including RDP, Chimaera (Posada and Crandall, 2001), BootScan (Salminen et al., 1995), 3Seq (Boni et al., 2007), GENECONV (Padidam et al., 1999), MaxChi (Smith, 1992) and SiScan (Gibbs et al., 2000). The detected recombination events were required to have a Bonferroni corrected P-value < 0.05 and to be supported by topological evidence and at least two different methods. Two recombination events detected only by MaxChi that could explain the significant tree differences between *EF-1α*, *RPB2,* and *RPB1* were also accepted because they were well supported by the infraspecies phylogenetic analysis. Manual inspection and correction were carried out by checking whether the recombined region supported a tree topology (using the ML and BI methods implemented in the software) different from that based on the ML/BI tree based on the three housekeeping genes. To analyze if there were significant differences in the number of recombination events in the three housekeeping genes and the *FUB* gene cluster, a chi-square test was carried out against the null hypothesis that the number of recombination events should be proportional to the length of the analyzed regions.

### *Ka/Ks* and Selective Pressure Analysis

*Ka* and *Ks* were calculated between each pair of isolates from *Foc* and 3Fo using DNAsp v6.12.01, and the means of the *Ka/Ks* values of all of the genes were compared by one-way ANOVA implemented in SPSS v22 (IBM, New York).

Selection analysis of branches and amino acid sites was carried out using algorithms implemented in the web server Datamonkey 2.0 (Weaver et al., 2018). Isolates with introgressed outgroup genetic material were excluded from the analysis of the corresponding recombinant genes. Branch-site model-based methods aBSREL (Smith et al., 2015) and BUSTED (Murrell et al., 2015) were carried out to test whether the branches leading to R1, R4 and 3Fo had been subjected to positive selection. ML-based methods FEL, SLAC (Kosakovsky Pond and Frost, 2005) and MEME (Murrell et al., 2012) were performed for selective pressure analysis of individual sites.

## Results

### Genetic Diversity of *Foc* Based on 12 FUB Genes and Three Housekeeping Genes

CDSs of three housekeeping genes (*EF-1*α, *RPB1*, and *RPB2*) and 12 *FUB* genes with a total length of 33,168 bp were subtracted from the assembled genome sequences of 16 race 1 group (R1) isolates and 18 race 4 group (R4) isolates (**Table 1**). A total of 1,505 single-nucleotide variations were detected in the CDSs (**Table S1**), including 1,464 biallelic and 41 triallelic loci. Of the 1,505 variations (1,546 mutations), 1,228 mutations were synonymous, 309 were nonsynonymous, and the remaining nine mutations were not assigned (explained in the *Materials and Methods* section). No highly deleterious mutation that caused an open reading frame shift or protein truncation was detected in any of the 15 genes in any isolate.

A higher level of genetic diversity was identified in R1 than in R4. Based on the 15 genes, 12 different genotypes were identified from the 16 R1 isolates, and nine different genotypes were identified from the 18 R4 isolates (**Figure S1**). More group-specific intragroup variations were detected in R1 than in R4 on all genes except *FUB5* (**Figure 1A** and **Table S1**). A total of 71 intragroup variations in *FUB* genes were shared between the two groups, which is most likely a sign of horizontal transfer between the two groups rather than convergent evolution. No shared intragroup variation was discovered in the three housekeeping genes. In total, 298 variations (51 nonsynonymous and 247 synonymous) were identified as intergroup variations in the 15 genes. The nucleotide diversity (*Pi*) of the R1 was generally higher (>2-fold and on average 6.43-fold) than that of the R4 in all genes except *FUB5*, on which R1 had lower (0.82-fold) *Pi* than R4. The *Pi* in the *FUB5* gene of R4 was significantly higher (on average 5.35-fold, *p* < 0.001) than the *Pi* in any other gene of the same group. The haplotype diversity of R1 is higher than that of R4 for all genes, including *FUB5*.

As shown in **Table S1**, the *Pi* in the *FUB* genes of both R1 and R4 is significantly (on average 7.5- and 11.1-fold, respectively, *p* < 0.01) higher than that in the three housekeeping genes. Differences in *Pi* could be the result of the combined effect of different selective pressures and different amounts of HGT, but differences in the number of synonymous variations were supposed to be affected by only HGT. According to the results (**Table S1**), the number of synonymous substitutions per synonymous site (*Ks*) between R1 and R4 was significantly (*p* < 0.01) higher for *FUB* genes than for the three housekeeping genes. As shown in **Figure 1B**, significantly higher nucleotide diversity of silent variations (*PIsi*) (*p* < 0.01) was observed in 77.3%, 45.7%, and 76.0% of the regions of the *FUB* gene cluster than in the three housekeeping genes in R1, R4, and *Foc*, respectively. The observed high *Ks* and *PIsi* in *FUB* genes should indicate introgression of outgroup genetic material into the *FUB* genes in either or both group(s).

### Infraspecies Phylogeny Analysis

Molecular data on the 15 genes were used to infer the infraspecific phylogeny of *Fo*. To reveal the phylogenetic relationships of the sequenced *Fo* isolates and four published *Fo* isolates, Fo4287, Fo26406, Fo47, and Fo25433, maximum likelihood (ML) and Bayesian inference (BI)-based phylogenetic methods were applied on concatenated nucleotide sequences of *EF-1α*/*RPB1*/*RPB2* and 12 *FUB* genes with Fv7600 as the outgroup. As shown in **Figure 2A and B**, phylogenetic trees obtained by different phylogenetic methods on the same gene set were highly consistent, and in phylogenetic trees based on both concatenated sequences, isolates of the same VCGs were clustered into the same clades. However, the phylogenetic trees based on the *EF-1*α/*RPB1*/*RPB2* gene set were poorly supported [significantly worse by the Shimodaira–Hasegawa (SH) test at the 1% level] by the 12-*FUB*-gene set and *vice versa*. In the trees based on the three housekeeping genes, four isolates (JB255, JB553, Race1-MW2, and Race1-MW40) were clustered in the R1, but in the 12-*FUB*-gene cluster, they clustered out of R1 and formed a monophyletic group with Fo4287, Fo26406, and Fo47, which are three nonbanana *Fo* isolates (referred to as the 3Fo group). The phylogenetic relationship of R1 isolates and R4 isolates based on *EF-1α*/*RPB1*/*RPB2* should be robust, since any recombination signal was seldom detected in R1 and R4 isolates on these three genes (see the following section).

To check the difference in the phylogeny of the 15 genes, ML and BI-based methods were applied to each of the 15 genes for all of the above accessions. In trees based on both concatenated sequences and most genes, the nonbanana isolate Fo25433 was highly supported as belonging to R1. As shown in **Figure S2**, contradictory branches were widely observed in the 15 gene trees. The results of SH tests showed that the molecular trees based on most genes were significantly different (*p* < 0.05, **Figure 2C**), and only *EF-1*α and *RPB2* did not reject each other (*p* > 0.05 by SH test), but both rejected the tree based on *RPB1*, showing that at least one HGT event occurred in the three genes. For the trees based on *EF-1*α and *RPB2*, the tree topology supports the idea that the 3FO diverged from the common ancestor of R1 and R4, while the tree based on *RPB1* supported that 3Fo and R1 shared a more recent common ancestor than with R4, which is also supported by phylogenetic trees based on both concatenated gene sets (**Figures 2A, B**).

Considering the widely present topological differences among gene trees, a method named branch support rate in randomly sampled segments (BSRRSS) was used to infer a more reliable infraspecies tree and assess the support rate of controversial branches. Randomly sampled 1- and 2-kbp segments from the CDSs of the 15 genes were both applied in this study and produced similar results. As shown in **Figure 2D**, in the branches best supported by randomly sampled 1-kbp (2-kbp) gene segments, the monophyly of R1 and the monophyly of R4 were supported by 12% (15%) and 86% (91%) of sampled segments, respectively. The monophyletic clade compromising R1 and 3Fo was supported by 31% (36%) of sampled 1-kbp (2-kbp) segments, and the ratios of sampled 1-kbp (2-kbp) segments supporting the monophyly of R1 and R4 and the monophyly of 3Fo and R4 were 15% (15%) and 4% (2%), respectively. When most recombinant R1 and R4 isolates identified in the following section were excluded from the analysis, the support for the monophyly of R1 and 3Fo was only slightly increased to 35% (38%), while the support for monophyly of R1 increased to 77% (81%). Considering that at most four loci with different lengths were sampled, the support for the monophyletic clade including R1 and 3Fo is not robust based on the 15 genes according to the BSRRSS test method. In the BSRRSS analysis, most 1- and 2-kbp gene segments did not contain phylogenetic signals supporting either monophyletic phylogeny or polyphyletic phylogeny of *Foc*, indicating that the differentiation of R1, R4, and 3Fo should have occurred in a short period, leaving only a weak phylogenetic signal in the genes.

### Enrichment of HGT Events for *FUB* Genes

**Figure 3:**
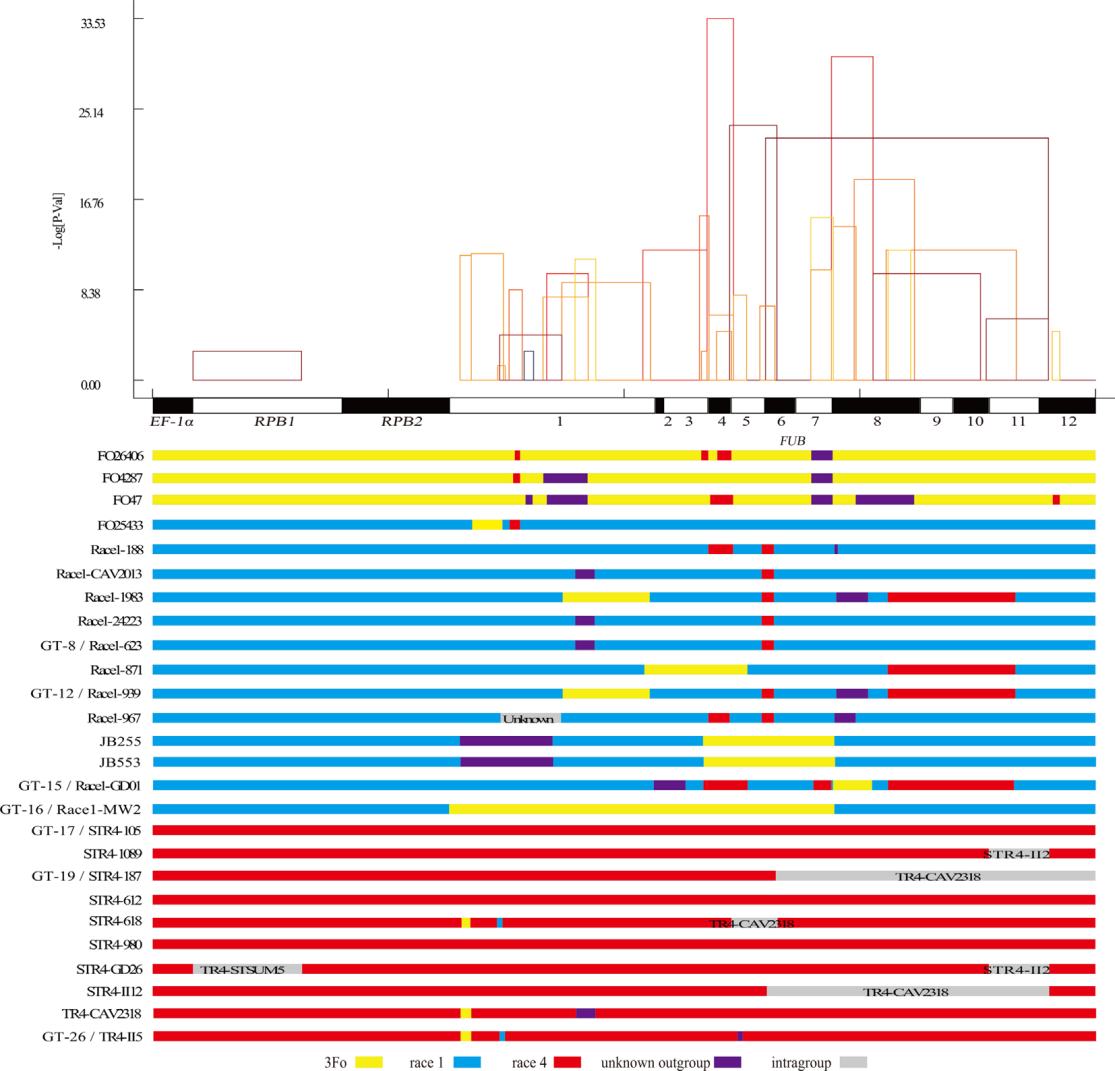
Distribution of inferred recombination events in the 15 genes. Top panel: each rectangle represents an inferred recombination event, and the horizontal coordinates of the two vertical edges of the rectangles indicate the inferred most likely recombination points of the recombination event by RDP4. The height (vertical axis) of each rectangle is the lg (*p* value) of the recombination event inferred by the algorithm implemented in RDP4. Bottom panel: introgressed gene segments identified in the 26 different *Fo* genotypes. Gray bands indicate intragroup recombination events in R1, R4, or 3Fo, and the name of putative source isolate (most likely the isolate which is the most similar to the source isolate) was shown on the graph.

According to the above results, HGT should have played a role in producing some of the *Foc* genotypes observed in this study, such as the four isolates JB255, JB553, Race1-MW2, and Race1-MW40. To reveal the distribution of recombination events that led to the 15 analyzed genes in the *Fo* isolates, seven different methods implemented in RDP4 were applied, and the topology in **Figure 2D** was assumed to be the true infraspecies tree in the process. Three recombination events were detected to have occurred in the three housekeeping genes. Two of the events were supported by only one method and were HGTs of the outgroup (which diverged earlier than the divergence of R1 and R4) *EF-1*α and *RPB2* genes into the 3Fo isolates, which could explain the conflict between the phylogenetic trees based on *EF-1*α, *RPB2*, and *RPB1*. The third recombination was identified within the R4 with support from two methods and involved HGT of partial *RPB1* of the TR4-STSUM5-like isolate into STR4-GD26. As shown in **Figure 3**, a significantly greater number of well-supported (detected by at least two methods and passing manual inspection) recombination events (14.7-fold, *p* < 0.001) were detected in the *FUB* gene cluster (1.41 recombination/kbp) than in the three housekeeping genes (0.096 recombination/kbp) in the *Fo* isolates. The recombination detection methods were supposed to detect only relatively large recombinant regions (Martin et al., 2015), and there should be an undetected recombination because the *PIsi* values were significantly higher in the *FUB* genes than in the three housekeeping genes in more regions (**Figure 1B**).

As shown in **Figure 3**, bottom panel, many more outgroup genetic materials were transferred into R1 than into R4, which partially explained why R1 had a much higher genetic diversity than R4. Large regions of horizontal gene-transferred outgroup genetic materials were observed in the *FUB* genes in Race1-MW2, Race1-MW40, JB553, and JB255. A recombination region that involved the horizontal transfer of ancient outgroup genetic material in *FUB5* was detected in II5 and five other R4 isolates, which should explain why the R4 had higher genetic diversity in only *FUB5*. In two R1 isolates, JB255 and JB553, which have lost their pathogenicity towards banana, *FUB1* was identified to be recombined with an outgroup isolate.

### Selective Pressure on *Fo* Genes

**Figure 4:**
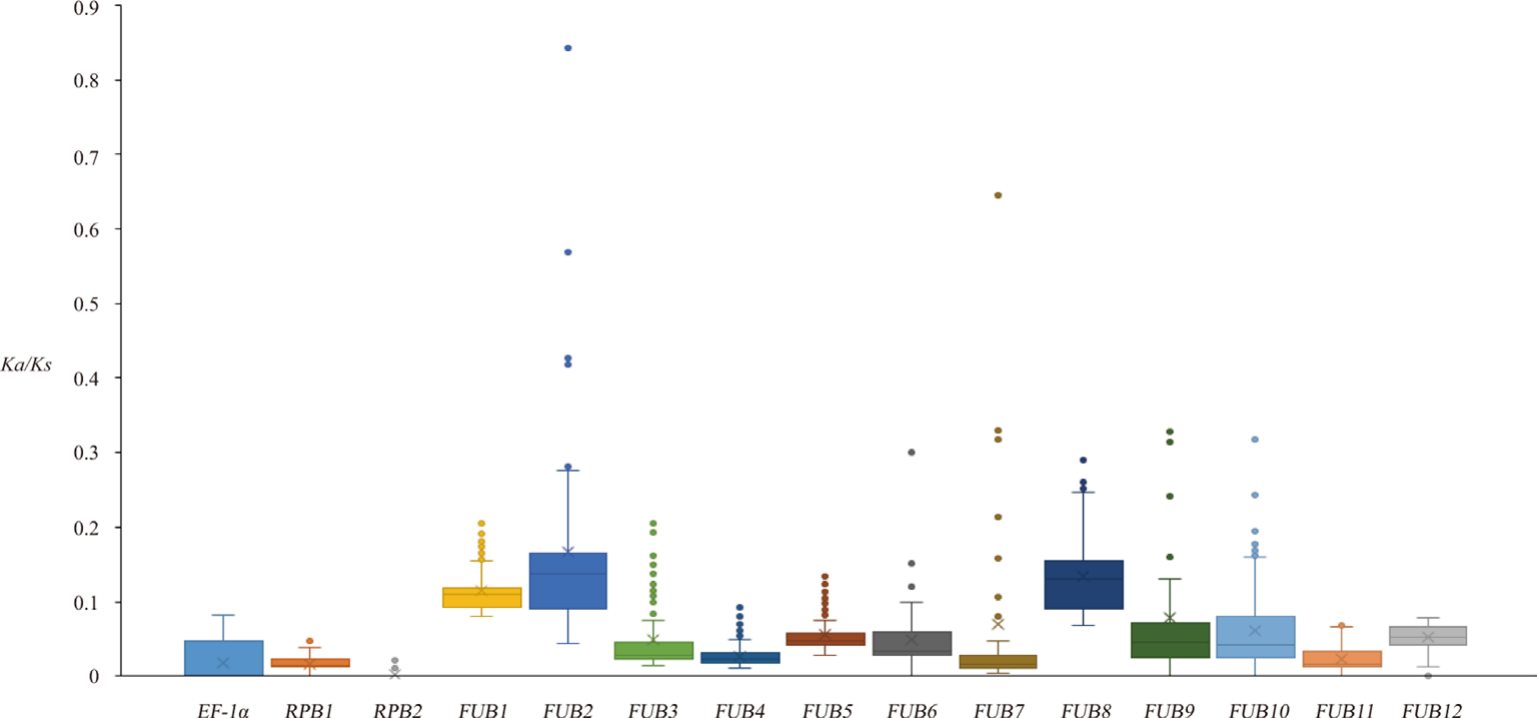
Boxplot of *Ka/Ks* distribution between *Fo* isolates based on the 15 genes. The mean *Ka/Ks* value is indicated by the small crosses on the graph.

The average *Ka*/*Ks* values for the 15 genes over different *Fo* isolates suggested that most coding loci on the 15 genes should have been subjected to negative selection (**Table S1**), because all of these values were much smaller than 1. The *FUB* genes other than *FUB11* had a significantly higher mean *Ka*/*Ks* values than each of the three housekeeping genes (**Figure 4**). The largest average *Ka*/*Ks* value was observed for *FUB2* (0.166 ± 0.122), and the *Ka*/*Ks* values between some isolates from 3Fo and R4 were as high as 0.84, suggesting that many loci of this gene could have been subjected to neutral evolution in some branches.

Branch-site models were applied to test for positive selection on the branches leading to R1, R4, and 3Fo groups. Positive selection was not identified in any of the tested branches for the three housekeeping genes and 12 *FUB* genes. Site analysis was carried out to test whether there were any codons subjected to positive or negative selection on the *FUB* genes. As shown in **Table S2**, 173 negatively selected amino acid sites but not a single positively selected site was detected (*p* < 0.05 by at least two methods) on the 12 *FUB* genes, showing that the translations of analyzed genes were mainly subjected to negative selection or neutral selection.

## Discussion

FA is an important virulence factor in *Fo* and other *Fusarium* species that are associated with the wilt symptom in banana and other plants (Li et al., 2013; Dong et al., 2014; Singh et al., 2017; Ding et al., 2018). Considering the function of FA involved in interactions with the host and environmental microorganisms (Brown et al., 2015), revealing the evolution characteristics of the *FUB* genes could help us understand how *Fo* isolates have evolved to accommodate different host types and environments. Indeed, in this study, we demonstrated that the genetic diversity and phylogeny of *Foc* isolates did have some specific characteristics in the 12 *FUB* genes compared with the housekeeping genes.

Our results showed that all 12 *FUB* genes in *Foc* were mainly subjected to negative selection according to selection pressure analysis (**Figure 4**) and that not a single highly deleterious mutation was identified on any of the 12 *FUB* genes in any isolate. The conservation of the *FUB* genes observed in this study suggested most of the FUB genes were functionally important, consistent with the report that FA is essential for the virulence of *Foc* against banana (Ding et al., 2018). Consistent with the report that the functions and importance of the 12 *FUB* genes are different (Brown et al., 2015), significant differences in selective pressure were found among the 12 *FUB* genes according to *Ka*/*Ks* analysis. The function of *FUB2* is unknown and seemed to be unimportant in deletion analysis (Brown et al., 2015); however, the *Ka*/*Ks* analysis suggested that *FUB2* was conserved in some phylogenetic branches, though it has the highest average *Ka*/*Ks* value and had probably been subjected to neutral evolution during the divergence of 3Fo and R4. Additionally, no single fixed variation was observed in *FUB2* between R1 and R4 (**Table S1**), which suggested that it could also have a function in some unknown circumstances. *FUB11* (FA transporter) and *FUB7* [*O*-acetylhomoserine (thiol-)lyase] are important in transporting FA from the intracellular space to the extracellular space and in FA production, respectively (Brown et al., 2015), and in this study, *FUB11* and *FUB7* were shown to be the most conserved of the 12 *FUB* genes by the *Ka*/*Ks* analysis. *FUB1* has been known as the key gene in the gene cluster and encodes a PKS, and a recombinant *FUB1* gene including a partial gene from an unknown outgroup isolate was observed in both JB255 and JB553, which have lost their pathogenicity towards banana. The correlation between the two facts deserves further experimental verification.

Significantly higher genetic diversity was observed in the *FUB* genes than in the three housekeeping genes, which is not explained by the difference in selective pressure. According to our analysis, the high genetic diversity of the *FUB* genes was mainly derived from significantly enriched introgression of outgroup genetic materials into the *Foc* groups, as shown in **Figures 1B** and **3**. In other words, recombination at *FUB* genes could have been positively selected, significantly enlarging the gene pool in *Foc* and other *formae speciales* of *Fo*. Because mutations in *FUB* genes have been mainly reported to affect the regulation and efficiency of FA production in *Fusarium* isolates (Brown et al., 2015), some recombination events should have the ability to change the pattern of FA production in *Fo* isolates. Considering that FA is involved in the interaction of *Fusarium* isolates with both plant hosts and environmental microorganisms (Brown et al., 2015; Bohni et al., 2016; López-Díaz et al., 2018), when the plant host and living environment are relatively stable, the *Foc FUB* genes should be mainly subjected to negative selection. However, when *Foc* isolates were brought into a new environment, recombination of the *FUB* genes with local *Fo* lineages or even other *Fusarium* species (ancient outgroup genetic materials were identified in some *Foc* isolates) could have a positive effect on their adaption, and this process is supposed to save much more time than developing new beneficial mutations. Several recombination events were also supposed to have occurred at the *SIX* genes, which encode the only identified family of effectors in *Fo*, as reported by Czislowski et al. (2018). These results suggested that recombination enrichment might be a common phenomenon for genes involved in pathogen–host interactions or interactions with environmental microorganisms in *Fo*; however, this hypothesis still needs further verification.

The previously inferred polyphyletic phylogeny (Fourie et al., 2009; Czislowski et al., 2018) of race 1, race 4, and *Foc* is not sufficiently robust according to our analysis. Polyphyletic phylogeny of *Foc* was inferred based on the concatenated sequence of *EF-1*α/*RPB1*/*RPB2* in the study of Czislowski et al. (2018), and the inferred infraspecies tree also supported that race 1 and race 4 should be two monophyletic groups. However, as shown in our results based on individual genes, only the gene tree based on *RPB1* supported the polyphyletic phylogeny of analyzed *Foc* isolates, while *EF-1α* and *RPB2* supported the opposite case, which indicated that the phylogenetic inference based on the concatenated sequence could have resulted in a well-supported (by bootstrap or posterior probability) but incorrect tree (Heled and Drummond, 2010). Moreover, recombination analysis suggested that a recombination event had occurred at RPB1 or that two separate recombination events had occurred at *EF-1α* and *RPB2*, respectively, in the 3Fo group, depending on which infraspecies tree is accurate. In addition, in the study of Czislowski et al. (2018) and our study, introgression of outgroup genetic materials was observed at both the *SIX* genes and the *FUB* genes in both R1 and R4 isolates, which could easily render gene trees supporting polyphyletic phylogeny of R1 and R4 similar to that discovered in a previous study (Fourie et al., 2009), although the difference in the results could also be caused by the difference in *Foc* isolates used. The BSRRSS analysis in this study showed that the polyphyletic phylogeny of *Foc* was supported by <40% of the regions of the 15 analyzed genes and that R1, R4, and 3Fo should have diverged within a short time period, which made the phylogeny signal representing the true intraspecific phylogeny relatively weak and easily covered by HGT events. This study also supported that Fo25433, which is a pathogen detected on cotton, should have originated from *Foc* race 1, and the infraspecies tree in the study of Czislowski et al. (2018) also suggested that many *formae speciales* of *Fo* should have originated from the *Foc* race 1. Considering the limited number of sequenced genes and the HGT commonly observed in all the studies, to achieve a highly reliable infraspecific phylogeny tree for *Fo*, it is necessary to sequence more genome regions of more isolates and resolve the complex recombination events in sequenced isolates first.

In conclusion, this study showed that negative selection on a majority of amino acids and enriched HGT events are the main evolutionary characteristics of the 12 *FUB* genes. Though no positive selection signal on any gene or amino acid has been detected in this study, the genetic diversity on the 12 *FUB* genes was greatly increased due to significantly enriched HGT events, suggesting some of the natural mutations could have an adaptive effect and positively selected. But such mutations were not detected in this study, either because they are too limited in number compared with synonymous mutations or because they do not exist in the coding regions. Infraspecies phylogeny analysis in this study suggested that *Foc* race 1, *Foc* race 4, and some other *Fo* f. sp. should have diverged in a short time, and the previously suggested polyphyly of *Foc* still needs more evidence.

## Author Contributions

SL, BW, and SXL analyzed the data and wrote the manuscript; ZS, RL, GY, CL and XG conceived and designed the experiments.

## Conflict of Interest Statement

The authors declare that the research was conducted in the absence of any commercial or financial relationships that could be construed as a potential conflict of interest.
