# Supplementary figures and images for: Genetic Diversity in FUB Genes of Fusarium oxysporum f. sp. cubense Suggests Horizontal Gene Transfer
Source: Front Plant Sci. 2019 Sep 4;10:1069. doi: 10.3389/fpls.2019.01069 (PMC6738028; doi:10.3389/fpls.2019.01069)

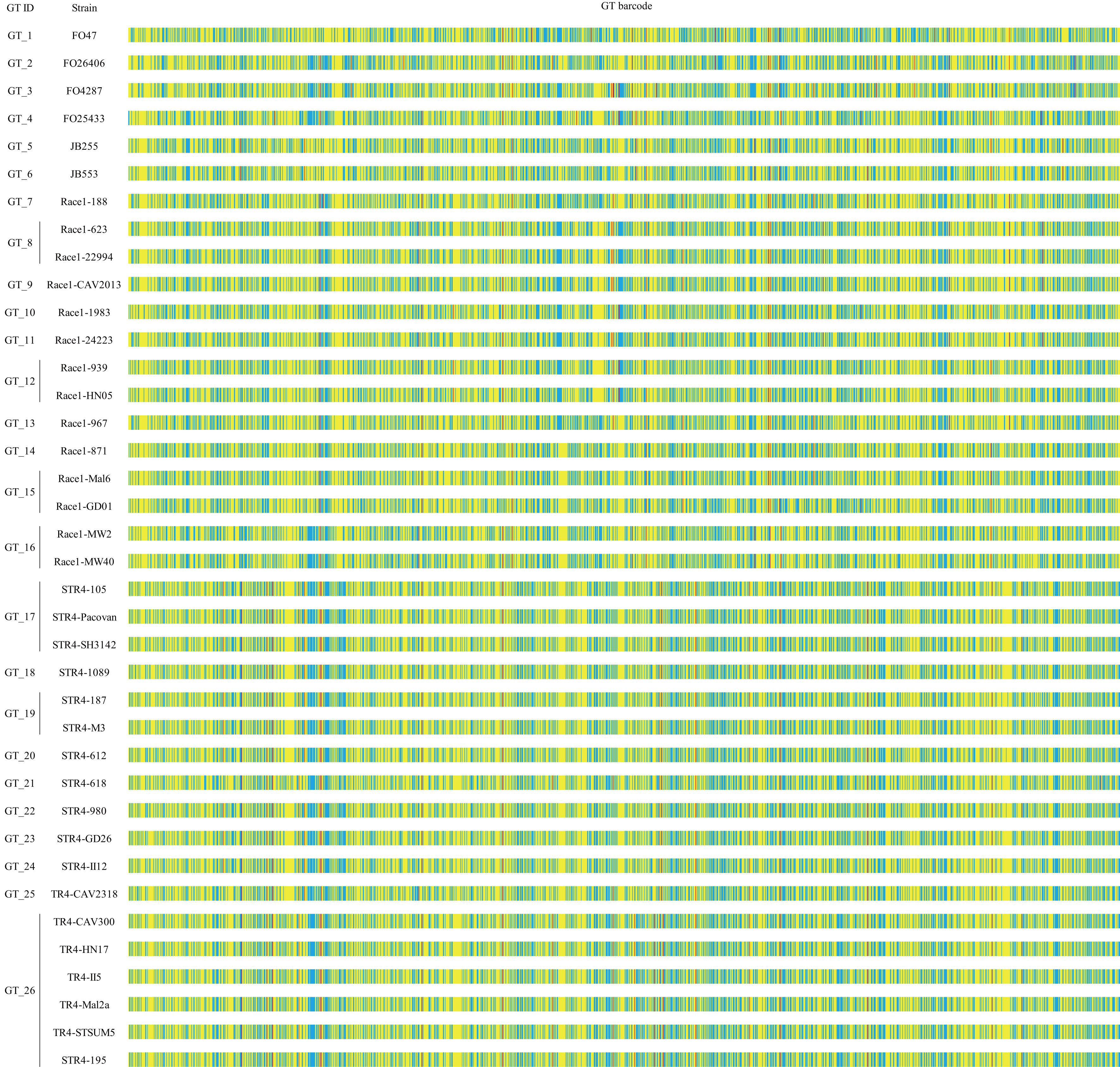

Supplement: Supplementary file 4 [file Image_1.tif]

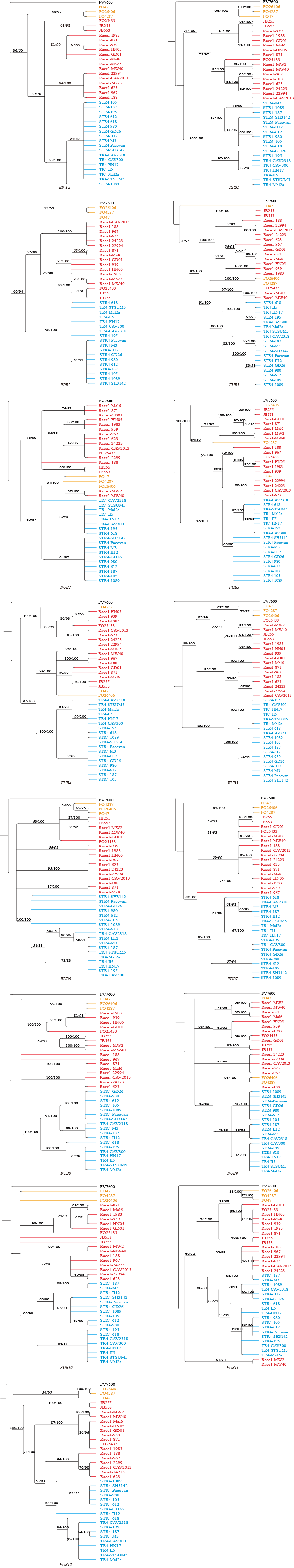

Supplement: Supplementary file 5 [file Image_2.tif]
